# Region-Specific Defects of Respiratory Capacities in the *Ndufs4*(KO) Mouse Brain

**DOI:** 10.1371/journal.pone.0148219

**Published:** 2016-01-29

**Authors:** Ernst-Bernhard Kayser, Margaret M. Sedensky, Philip G. Morgan

**Affiliations:** 1 Center for Developmental Therapeutics, Seattle Children's Research Institute, Seattle, Washington, United States of America; 2 Department of Anesthesiology and Pain Medicine, University of Washington, Seattle, Washington, United States of America; UMASS-Amherst/Tufts University School of Medicine, UNITED STATES

## Abstract

**Background:**

Lack of NDUFS4, a subunit of mitochondrial complex I (NADH:ubiquinone oxidoreductase), causes Leigh syndrome (LS), a progressive encephalomyopathy. Knocking out *Ndufs4*, either systemically or in brain only, elicits LS in mice. In patients as well as in KO mice distinct regions of the brain degenerate while surrounding tissue survives despite systemic complex I dysfunction. For the understanding of disease etiology and ultimately for the development of rationale treatments for LS, it appears important to uncover the mechanisms that govern focal neurodegeneration.

**Results:**

Here we used the *Ndufs4*(KO) mouse to investigate whether regional and temporal differences in respiratory capacity of the brain could be correlated with neurodegeneration. In the KO the respiratory capacity of synaptosomes from the degeneration prone regions olfactory bulb, brainstem and cerebellum was significantly decreased. The difference was measurable even before the onset of neurological symptoms. Furthermore, neither compensating nor exacerbating changes in glycolytic capacity of the synaptosomes were found. By contrast, the KO retained near normal levels of synaptosomal respiration in the degeneration-resistant/resilient “rest” of the brain. We also investigated non-synaptic mitochondria. The KO expectedly had diminished capacity for oxidative phosphorylation (state 3 respiration) with complex I dependent substrate combinations pyruvate/malate and glutamate/malate but surprisingly had normal activity with α-ketoglutarate/malate. No correlation between oxidative phosphorylation (pyruvate/malate driven state 3 respiration) and neurodegeneration was found: Notably, state 3 remained constant in the KO while in controls it tended to increase with time leading to significant differences between the genotypes in older mice in both vulnerable and resilient brain regions. Neither regional ROS damage, measured as HNE-modified protein, nor regional complex I stability, assessed by blue native gels, could explain regional neurodegeneration.

**Conclusion:**

Our data suggests that locally insufficient respiration capacity of the nerve terminals may drive focal neurodegeneration.

## Introduction

Mitochondrial disorders comprise the most common group of inborn errors of metabolism, with an estimated prevalence of 1 in 5,000 live births [1]. Many of these are devastating, progressive, neurodegenerative diseases for which there are currently no treatments. Leigh Syndrome (LS), also termed subacute necrotizing encephalopathy, is the most common infantile mitochondrial disease [2–4]. The signs and symptoms of LS include failure to thrive, hypotonia, acidosis, ataxia, visual and auditory deficiencies, and seizures. Neuropathological features of LS include region-specific neuronal loss, gliosis, white matter spongiosis and vasculo-necrotic lesions [5, 6]. The basal ganglia, brain stem and cerebellum appear preferentially vulnerable to mitochondrial dysfunction [4, 7] Most patients exhibit symptoms as infants and die within a year of diagnosis. The varied symptoms of LS largely result from profound and progressive region-specific pathology within the CNS [8, 9], a fundamental feature shared by other common diseases linked to mitochondrial dysfunction (Parkinson’s, Alzheimer’s, Huntington’s).

Defects in complex I of the electron transport chain comprise the majority of known causes of LS [10]. Mutations in the non-catalytic 18kD subunit of complex I encoded by the nuclear gene *Ndufs4* have been identified in five patients with a severe form of LS; all died by 22 months of age [11]. Symptoms in these patients included failure to thrive, hypotonia, encephalopathy, cardiomyopathy, and visual defects. All patients displayed focal CNS degeneration that affected bilateral basal ganglia, brainstem and cerebellar lesions. A further 13 patients with LS have been described with similar presentations, *i*.*e*. focal pathology and loss of NDUFS4 [7, 11–13]. In most patients, complete loss of NDUFS4 protein was identified, strongly supporting the use of a KO mutant (as opposed to only hypomorphic mutants) as a model to investigate the underlying mechanism leading to focal CNS degeneration.

Such a mouse model with a deletion of the second exon of the *Ndufs4* gene is available. The homozygote KO is lacking the NDUFS4 protein in all cells. The primary defect in complex I causes a progressive encephalomyopathy leading to death within 2 months and mirroring many aspects of human LS [8, 9] [14, 15], including focal damage to the CNS. Dysfunction within the CNS appears to be the driving factor for disease progression because KO of *Ndufs4* exclusively in brain cells causes largely the same phenotype as the whole body KO [9].

A puzzling feature of both LS and the mouse model is the regional variation in neurodegenerative susceptibility. In the mouse, all brain cells lack the NDUFS4 subunit of complex I, yet only a select few regions of the brain develop inflammation and eventual degenerate. The olfactory bulb, brainstem (especially the vestibular nuclei and the inferior olive) and the cerebellum (especially the caudal vermis) begin to show histologic defects by about day 35 of life, while the remainder of the mid- and forebrain are resistant to sequelae of loss of this protein. The link between mitochondrial dysfunction and region-specific neurodegeneration are not understood. Tissue specific factors must influence the response of the cell to the primary defect. It is possible that this leads to deterioration of mitochondrial function over time [16]; however, in LS there is a paucity of data to support this model [10].

We hypothesized that the local neurodegeneration in the olfactory bulb (OB), brainstem (BS), cerebellum (CB) may be a consequence of progressive loss of respiratory capacity in the neurons of the KO. We isolated mitochondria and synaptosomes from the susceptible regions of the CNS and compared their respiration to matching organelles from the degeneration-resistant/resilient “rest” (R) of the brain at age P25-P35 when the KO was still neurologically asymptomatic and at P45-P55 when significant CNS symptoms were apparent. We present evidence that the function of non-synaptic mitochondria remains stable while mitochondrial function within nerve terminals declines with age in the KO particularly in the degeneration prone regions of the CNS.

## Material and Methods

### Ethics Statement

All animal experiments were conducted following the recommendations in the Guide for the Care and Use of Laboratory Animals of the National Institutes of Health and were approved by the Animal Care and Use Committee of the Seattle Children’s Research Institute (IACUC protocol 13416). Every effort was made to minimize suffering.

### Animals

Mice were maintained on a standard rodent diet with 12 hours dark-light cycle at 22°C. Water and food was available ad libitum. The *Ndufs4*(KO) (*Ndufs4*Δ/Δ) mice needed to be generated by crosses of heterozygote parents (*Ndufs4*Δ/+) in a C57Bl/6 genetic background. Genotypes were determined by PCR. Since heterozygotes are healthy and have NDUFS4 protein levels [8] similar to the wild-type (*Ndufs4*+/+) both were used as genomic controls henceforth simply referred to as controls. Likewise, both male and female animals were used because disease relevant phenotypes were similar between genders [8, 9]. KO mice were kept with control littermates and received supplemental moistened food pellets and hydration gel when necessary. Mice were sacrificed by isoflurane overdose followed by decapitation. The brains were dissected into 4 regions: olfactory bulb (O), cerebellum (C), brainstem (B) and the remainder referred to as “rest” brain (R) consisting of midbrain and the entire forebrain except olfactory bulb. Two age groups of mice were studied: Young mice, postnatal age P25-35, before the reported occurrence of spongiforme lesions in the KO brains [9] and older mice, age P45-55 with clear symptoms of neuropathy such as clasping and ataxia in the KO [8, 9].

### Isolation of Brain Mitochondria

Nonsynaptic mitochondria from whole brain (without O) or from regions B, C, R were isolated based on the Percoll step gradient “method A” of Wang *et al*. [17] with the following modifications: The Percoll concentration of the lower phase was increased to 15% w/v. Mitochondria were washed twice with Sucrose Washing Buffer, first containing1mg/ml defatted BSA, then without BSA.

### Mitochondria Respiration Assay

A Seahorse XF24 flux analyzer (Seahorse Bioscience, North Billerica, MA) was used adhering to the manufacturer’s technical brief “Analyzing microgram quantities of isolated mitochondria in the XF24 Analyzer”. Assay specific parameters: Complex I dependent respiration was fueled by 10mM of either pyruvate, glutamate or α-ketoglutarate supplemented with 5mM malate respectively with 5μg protein loaded per well. Complex II dependent respiration was determined with 2μg protein loaded per well and electron donor succinate (13mM) in the presence of the complex I inhibitor rotenone (2μM). Initial conditions were 500μl per well Mitochondrial Assay Solution (MAS, 1X: 70 mM sucrose, 220 mM mannitol, 10 mM KH2PO4, 5 mM MgCl2, 2 mM HEPES, 1.0 mM EGTA and 0.2% (w/v) fatty acid-free BSA, pH 7.2 at 37°C) complete with substrates. Drugs dissolved in MAS including substrates were successively injected: ADP (50ul 40mM pH7.2), oligomycin (55ul 25μg/ml), carbonylcyanide-p-trifluoromethoxy-phenylhydrazone (FCCP) (60μl 50μM), antimycin A (65μl 40μM). Measurement program: Calibrate, wait 10min, 2x (mix 1min, wait 3min), 2x (mix1min, measure 3min), 4x (inject, mix 1min, measure 3min, mix 1min). Oxygen consumption rates (OCR) were analyzed in “point-to-point” mode where the output for each measurement step in the program is a time course of OCR data points. For each time point, OCR were corrected for background by deducting the mean of the control wells (without mitochondria) from each sample well (with mitochondria). For each well the mean value after antimycin A addition (non-mitochondrial OCR) was deducted from all previous measurements yielding mitochondrial specific rates for: State 3 (maximum value after ADP addition), state 4o (mean value after oligomycin addition), uncoupled (maximum value after FCCP addition). The optimal uncoupler concentration had been determined by titrating whole brain mitochondria with FCCP ranging from 1 to 15μM. Maximal response was observed between 5 to 10 μM FCCP. Subsequently 5uM FCCP was adopted for uncoupling of mitochondria from all regions). For complex I dependent substrates, state 3 rates were always higher than the subsequently recorded uncoupled rates. We therefore report state 3 since they not only represent the physiologically important capacity for oxidative phosphorylation but are also, in our protocol, a better estimate of the capacity to respire than uncoupled OCR.

Rates from > = 3 biological samples were compared by age and genotype using two-tailed Student’s t-test with α set to 5% employing the Holm-Bonferroni correction for multiple comparisons. Each biological sample (mitochondria preparation) is represented by the mean of 3–5 technical replicates.

### Isolation of Synaptosomes

Synaptosomes from regions O, B, C, R were isolated according to Choi *et al*. [18] with minor modifications. All steps of the preparation were performed at 4°C or on ice with ice cold solutions. Brain tissue was rinsed with “sucrose medium” (320 mM sucrose, 5mM Tris, 1 mM EDTA, 0.25 mM dithiothreitol, pH 7.4) followed by Dounce homogenization, 8 strokes each with the loose and then the tight fitting pestle. R was homogenized in 6ml of sucrose medium, while 3ml was used for O, B, C each. The homogenates were centrifuged 1000g for 10min. Each supernatant was layered on top of a preassembled Percoll density step gradient consisting of 4ml 23%, 3ml 10% and 3ml 3% Percoll in sucrose medium. After 10min centrifugation at 32500g in a Sorvall SS-34 rotor a turbid band of synaptosomes was recovered in 2ml from the interface between 23% and 10% Percoll. Synaptosomes were washed by adding 4ml of sucrose medium and 10min centrifugation at 15000g. The pellet was resuspended in 1.5ml sucrose medium, transferred to a microfuge tube and spun again to yield the final synaptosomal pellet which was resuspended in less than 100μl sucrose medium. Protein concentrations were measured with the DC microplate assay (Bio-Rad Laboratories, Hercules CA).

### Synaptosome Respiration Assay

Respiration was measured with a Seahorse XF24 flux analyzer as described by Choi *et al*. (2011) [19] with minor modifications. To improve retention of synaptosomes Seahorse cell culture plates were double coated, first with polyethyleneimine (100μl/well, 1:15000 dilution in water, over night at room temperature) and then, after aspiration, additionally with geltrex (LifeTechnologie, Waltham, MA) (100μl/well, 1:100 dilution in water, at 37°C). Wells were loaded with 10–13μg protein dispensed in 100μl of ice cold “ionic medium” (20 mM HEPES, 10 mM D-glucose, 1.2 mM Na_2_HPO4, 1 mM MgCl_2_, 5 mM NaHCO_3_, 5 mM KCl, 140 mM NaCl, pH. 7.4). 5 technical replicates were plated for each synaptosomal preparation. Synaptosomes were attached to the bottom of the wells by centrifuging (3200g, 30min, 4°C). After substitution of the supernatant with 700μl/well incubation medium (3.5 mM KCl, 120 mM NaCl, 15mM glucose, 10mM Na^+^-pyruvate, 1.3 mM CaCl_2_, 0.4 mM KH_2_PO_4_, 1.2 mM Na_2_SO_4_, 2 mM MgSO_4_, 4 mg/ml fatty acid free bovine serum albumin, pH7.4 at room temperature) plates were loaded into the analyzer which was programmed to monitor oxygen consumption rates (OCR) and external acidification rates (ECAR) at 37°C for 19 measurement cycles each consisting of 30s mixing, 2min pause, 1min measuring. After 7 cycles under initial conditions, the ATP synthase inhibitor oligomycin (4ng/μl assay concentration) was injected to block respiration stimulated by oxidative phosphorylation. Three cycles later the protonophore uncoupler carbonylcyanide-p-trifluoromethoxy-phenylhydrazone (FCCP) (8μM assay concentration) was injected to stimulate maximal mitochondrial respiration. After 3 more cycles a mix of the complex I inhibitor rotenone and the complex III inhibitor antimycin A was injected to completely block mitochondrial respiration in order to determine non-mitochondrial oxygen consumption. Three cycles later, 4-aminopyridine (1mM assay concentration) was injected to increase synaptosomal ATP demand and thus stimulate glycolysis [18]. ECAR was recorded for 3 more measurement cycles. All injections were 75μl/well with the drugs dissolved in incubation medium.

Data reduction: Background wells (control wells without synaptosomes) with erratic OCR traces were removed from the background group. For each measurement the background mean was deducted from each sample well and OCR and ECAR were normalized to the amount of protein loaded. Then “non-mitochondrial respiration” (mean of 3 OCR measurements after injection of antimycin A and rotenone was subtracted from all the raw OCR rates to yield mitochondria specific rates. “Basal” respiration was then calculated as the mean OCR of the 3 cycles preceding the first drug injection. (During earlier cycles respiration had not reached steady state yet.) “State 4” was calculated as the mean OCR of three time points following oligomycin injection. The “capacity” to respire was the maximum OCR during 3 cycles after injection of FCCP. Maximal glycolytic activity was represented by the mean ECAR of the last 3 cycles.

After data from sample wells with erratic traces had been discarded the mean rates of the remaining (minimally 3) technical replicates for each preparation represented the rates for one biological replicate. The reported rates are mean ± SEM of at least 3 independent biological replicates. For each brain region separately, rates were compared by age and genotype using two-tailed Student’s t-test with α set to 5% employing the Holm-Bonferroni correction for multiple comparisons.

### SDS-PAGE and Western Blot

*General Procedure*: Standard SDS PAGE preparation according to Laemmli using 10% polyacrylamide gels [20]. All samples were denatured under reducing conditions for 15min 70°C in SDS sample buffer. Western blotting onto low fluorescence Millipore Immobilon FL, PVDF, 0.45μm membrane using Tris (25mM) glycine (148mM) transfer buffer with 20% methanol. Blocking and antibody incubations in TBS with 5% and 2% w/v fat free dry milk respectively. TBS-0.1%Tween20 was used for all washing steps.

*Mitochondria*: Fresh preparations were frozen and kept at -80°C until further use. For detection of HNE damage 40ug protein were loaded per lane. Primary antibody anti HNE-fluorophore rabbit polyclonal IgG (Calbiochem/EMD Millipore 393206, 1:2000) [21]. Secondary antibody HRP conjugated goat anti-(rabbit-IgG) (Santa Cruz SC-2054, 1:5000). Pierce ECL Western Blotting Substrate (Thermo Scientific #32106) was used to image immunoreactive bands by enhanced chemiluminescence. For densitometry film sheets were scanned at 600dpi and saved as 8-bit greyscale TIF files to be analyzed with ImageJ [22]. For mitochondrial samples the raw HNE signal was normalized as described in S2 Fig.

*Synaptosomes* Proteinase inhibitors (Sigma P3840) and 10x RIPA buffer were added to fresh synaptosomal preparations to a final concentration of 1x RIPA (1mM EDTA, 1% v/v Triton X-100, 0.1% w/v sodium deoxycholate, 0.1% SDS, 0.14M NaCl, 10mM TrisCl pH8.0) incubated at RT for 10min and stored at -80°C until further use. Synaptophysin and ATPase α were simultaneously detected in synaptosome samples (4μg/lane) with duplexed primary antibodies anti-synaptophysin YE269 rabbit monoclonal IgG (Abcam ab32127, 1:40000) and anti ATP5A 15H4C4 mouse monoclonal IgG2b (Abcam ab14748 1:40000) and duplexed secondary antibodies anti-(rabbit-IgG(H+L)) DyLight680 (goat polyclonal IgG, Thermo Scientific #35568, 1:10000) and anti-(mouse-IgG(H+L)) DyLight800 (goat polyclonal IgG, Thermo Scientific #35521, 1:10000). Near infrared fluorescence of the secondary antibodies was imaged with an Odyssey scanner (Li-Cor Biosciences, Lincoln, NE) with intensity settings of 3.5 and 5.0 for channels 700 and 800 respectively. For the detection of HNE-damage in 10ug/lane synaptosomal protein the same antibodies as described for mitochondria were used.

### Blue Native Gel Electrophoresis

BNG-PAGE was performed as published using digitonin (6:1 detergent:protein w:w) to solubilize mitochondrial supercomplexes [23]. Complex I in gel activity (IGA) staining (= diaphorase activity stain) as described previously [24].

## Results

### Function of non-synaptic KO mitochondria does not deteriorate systemically over time

**Fig 1:**
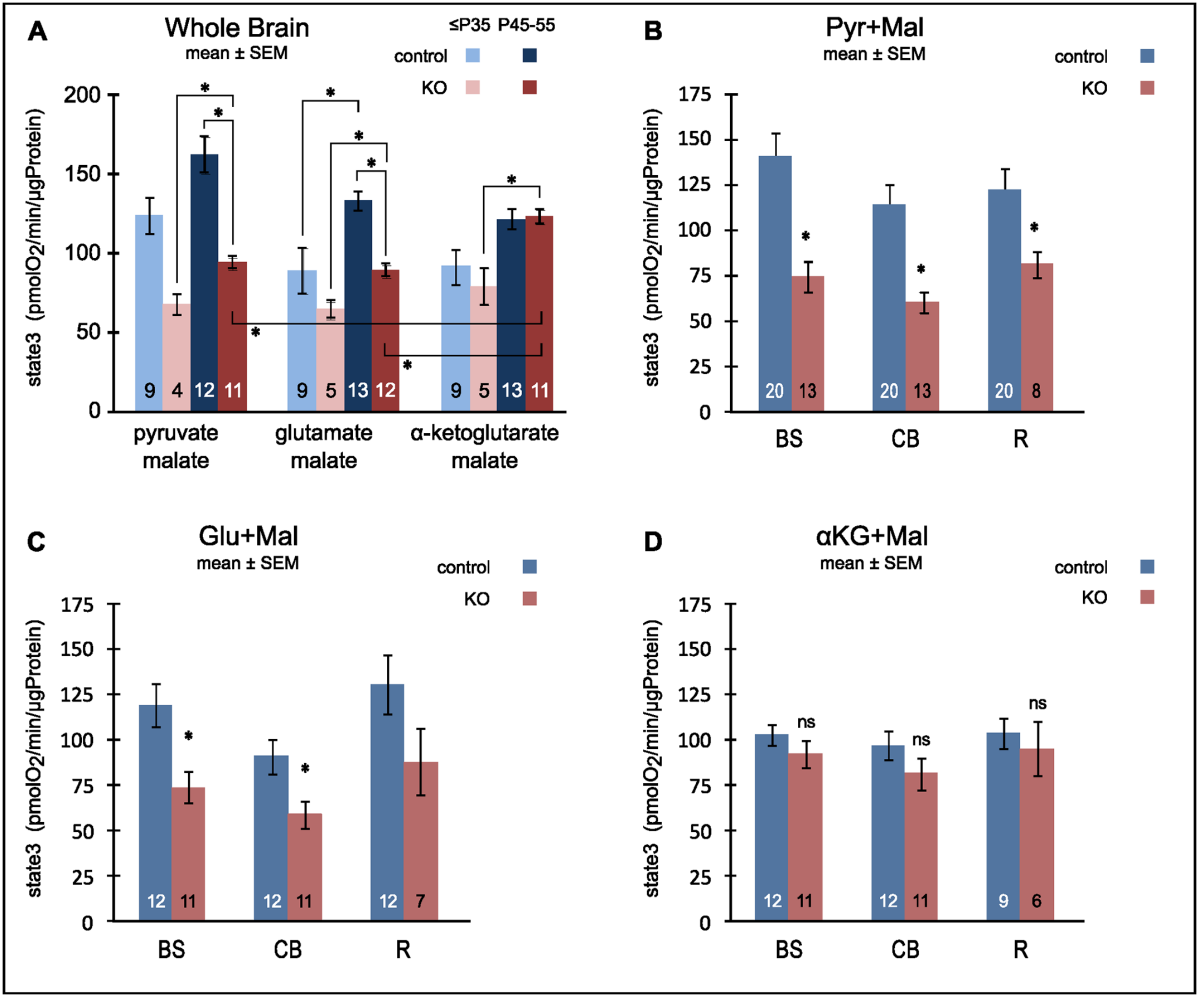
Complex I dependent capacity for oxidative phosphorylation of isolated (non-synaptic) mitochondria. (A) Maximal ADP-stimulated respiration (state 3) of mitochondria from whole brain fuelled with complex I dependent electron donor substrates. Note, while increase of oxphos capacity with age only reached statistical significance for controls powered with glutamate/malate, it becomes significant (3-way ANOVA P<0.001) for the aggregate of all substrates and all genotypes. (B,C,D) State 3 respiration for mitochondria isolated from brain stem (BS), cerebellum (CB) and remainder of brain (R). The complex I dependent electron donor substrates were pyruvate + malate (B), glutamate + malate (C) and α-ketoglutarate + malate (D). Olfactory bulb was excluded from both whole brain and R preparations. * denotes significant (α = 0.05) differences after Holm's correction for multiple pairwise comparisons.

We first studied the progression of mitochondrial dysfunction in *Ndufs4*(KO) in the whole brain over the time period during which KO animals show behavioral and histopathologic deterioration (Fig 1A). We did not detect any progressive worsening of complex I activity in either control or KO animals at the whole brain level. To the contrary, when all substrates were considered the capacity for state 3 respiration (oxidative phosphorylation) increased regardless of genotype.

As reported previously, complex I dependent activity of the KO was lower than controls when either pyruvate/malate or glutamate/malate were used as substrates [24]. This deficiency in the KO reached significance in the older cohort. When α-ketoglutarate/malate was used as substrate however, no difference was noted between control and KO oxidative phosphorylation at either age (Fig 1A). Moreover, in the older KO the residual activity is considerably higher than with the other complex I substrates (Fig 1A). The activities of all three electron donor combinations were fully inhibitable by rotenone confirming that they were entirely complex I-dependent.

Complex I independent respiration was tested by fueling mitochondria with the complex II electron donor succinate. (S1 Fig). State 3 respiration rates were statistically indistinguishable between genotypes and age groups. Thus, the diminished respiration with complex I dependent substrates in the KO is indeed due to loss of complex I activity and not caused indirectly by inhibition of electron transport downstream of complex I.

### Does mitochondrial function deteriorate regionally?

We next explored whether those areas of the brain that show the greatest injury late in development have worse complex I function than do relatively unaffected areas. Grouping our results by CNS region (Fig 1B, 1C and 1D) and including both P<35d and P45-65d ages, we found a significant difference in complex I-dependent state 3 capacities between control and KO animals in mitochondria from the vulnerable regions brainstem (BS) and cerebellum (CB) when pyruvate/malate (Fig 1B) or glutamate/malate (Fig 1C) were used as substrates. In the degeneration resilient “rest” of the brain (R = midbrain and forebrain without olfactory bulb) we also found diminished capacity in the KO. The difference between KO and control reached significance with electron donor pyruvate/malate but not with glutamate/malate. As noted for mitochondria from whole brain KO mitochondria of all brain regions respired normal when fueled with the complex I dependent substrate α-ketoglutarate/malate (Fig 1D). Over all the residual complex I dependent activity was not clearly distinct between vulnerable and resilient regions in the KO.

### Regional supercomplex stability

**Fig 2:**
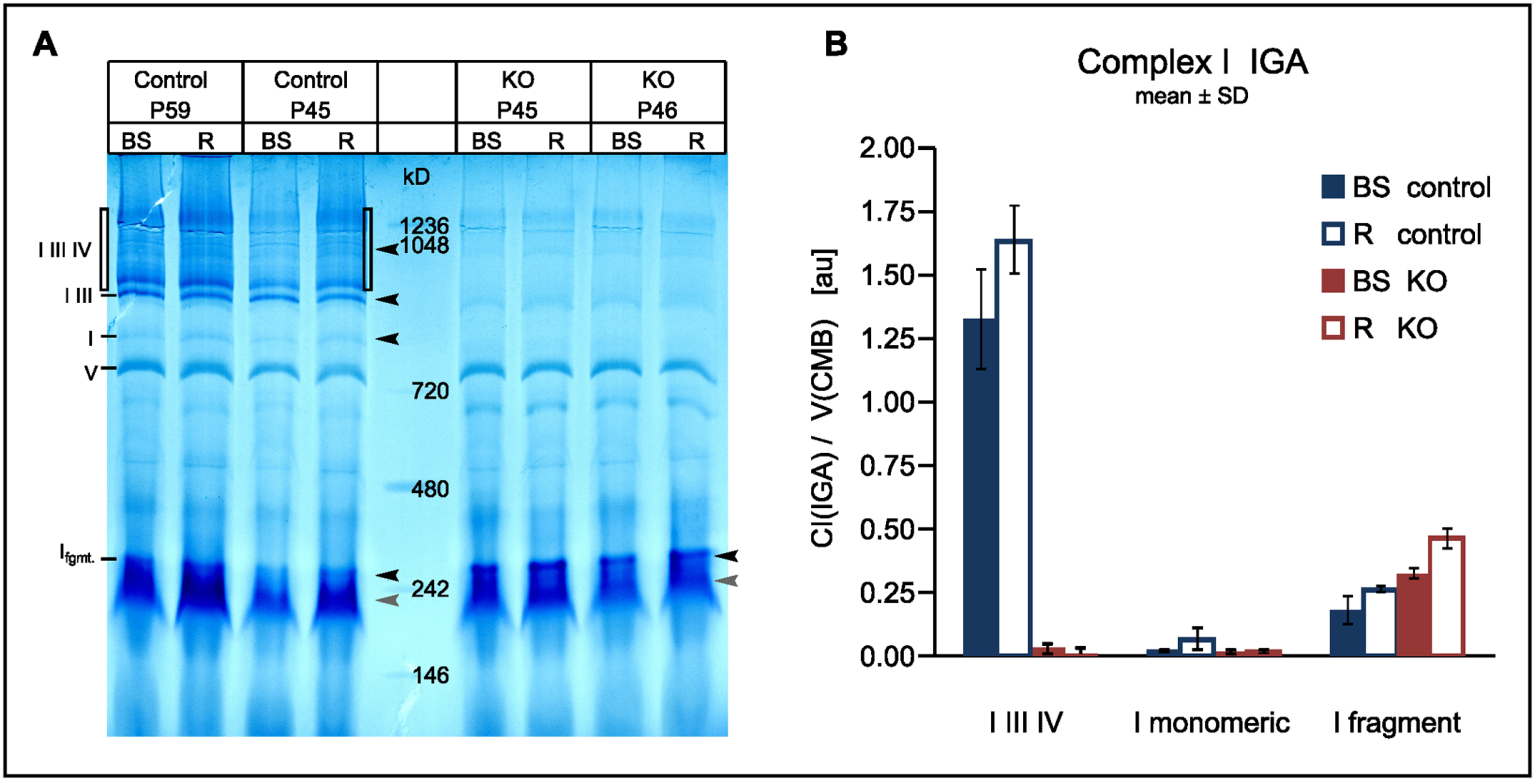
Stability of complex I is not increased in KO mitochondria from resilient "rest" brain. Blue Native PAGE of mitochondrial proteins from brainstem (BS) and “rest”brain (R) were electrophoresed under conditions which leave wildtype mitochondrial supercomplexes intact (A). Purpelish diaphorase activity staining revealed bands containing the NAD binding site of complex I (black arrow heads). All proteins were nonspecifically counterstained aqua blue with Coomassie Blue. Select landmark bands are labeled: supercomplexes containing complex I III and IV (I III IV), incomplete supercomplex (I III), solitary complex I (I), ATP synthetase (V), a low molecular weight fragment of complex I (I fgmt). (Strong diaphorase activity at grey arrow heads may be dihydrolipoamid DH). Densitometry results (B): Activity stain for the indicated complex I containing bands was normalized against the protein stain of the complex V band (serving as an internal loading control).

Intact mitochondria from the *Ndufs4*(KO) mice retain about half the normal capacity for complex I specific oxidative phosphorylation. In contrast, virtually no complex I electron transport activity can be measured once KO mitochondrial membranes have been disintegrated by sonication [8] or solubilized by cholate (our unpublished data). The lability of KO complex I activity led others to postulate that the non-catalytic NDUFS4 subunit is either necessary for stability [8] or for proper assembly of complex I [25]. We used blue native gels to investigate whether region-specific factors might stabilize KO complex I. For sample preparation the non-ionic detergent digitonin was chosen for its property to leave assembled supercomplexes intact. Diaphorase activity stain was used to track the NADH dehydrogenase activity of complex I. We found a dramatic loss of complex I containing supercomplexes in the CNS of the KO (Fig 2**)** regardless of whether the tissue was from affected (brainstem, BS) or unaffected (“rest”, R) parts of the brain. Likewise, solitary complex I, a minor band in controls, was also virtually absent in the KO. Only an incomplete fragment was found in the KO. Thus, no region specific effect on the stability of either solitary complex I or supercomplex could be detected.

### Progression of region-specific differences

#### Oxidative Phosphorylation

**Fig 3:**
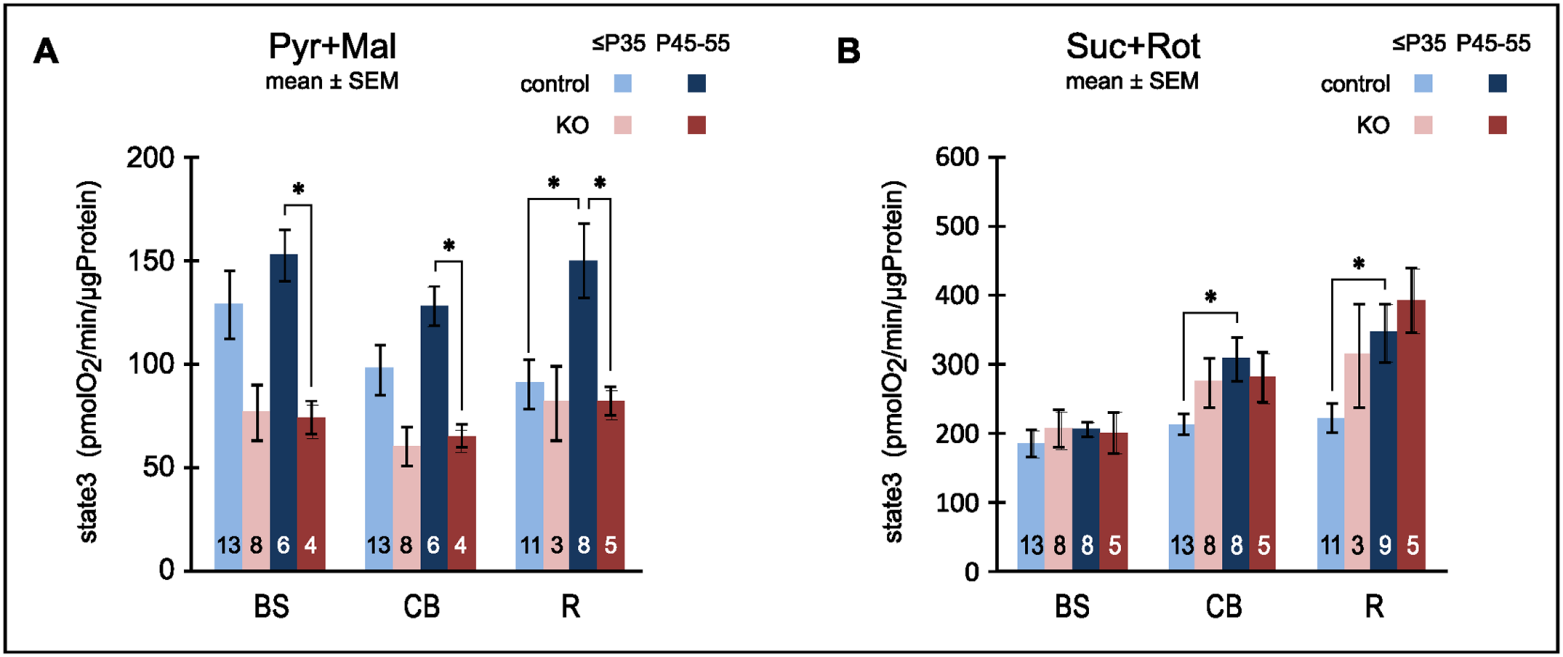
Capacity for oxidative phosphorylation (state 3 respiration) of isolated non-synaptic mitochondria by brain region and age group. A: Complex I-dependent state 3 respiration with pyruvate plus malate remained constant with age in the KO but tended to increase in the controls leading to significant capacity shortfalls in older KO mice in all brain regions tested: brain stem (BS), cerebellum (CB) and remainder of the brain excluding olfactory bulb (R). B: With complex I dependent electron transport blocked by rotenone, the capacity for state 3 respiration via complex II increased with age in the controls reaching significance in CB and R. In the KO capacity did not significantly increase with age but was already elevated in the young cohort. The number of biological replicates is stated inside the bars. * denotes significant changes at significance level (α = 5%) after Holm-Bonferroni correction for multiple comparisons.

Next we investigated whether state 3 respiration of non-synaptic mitochondria changes over time differently in affected and unaffected brain regions. We focused on the electron donor substrate pyruvate/malate because it has consistently elicited the largest differences between the genotypes. Complex I dependent state 3 respiration was expectedly lower in the KO than in control animals (Fig 3). This difference reached statistical significance in the older cohort for all regions. In line with the results for whole brain and contrary to our initial expectation, in the KO the absolute state 3 respiration did not worsen over time but the difference between control and KO increased. The relative loss of capacity may contribute to disease progression but cannot be the sole cause for focal neurodegeneration because it is equally observed in affected and unaffected brain regions.

Complex II dependent state 3 rates increased with age in both CB and in R in controls. In the KO animals, they remained constant with age in all regions (Fig 3B).

#### ROS Damage

**Fig 4:**
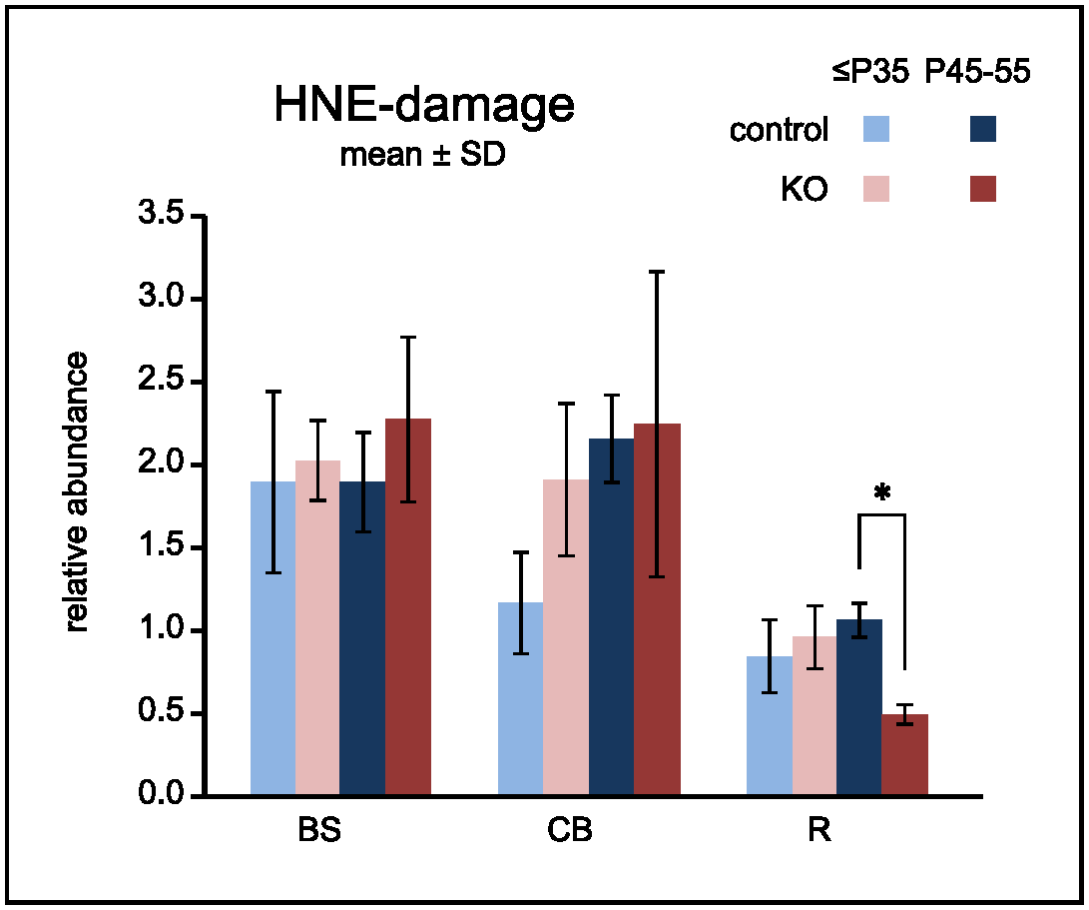
HNE damage to mitochondrial protein from vulnerable regions in KO is not elevated compared to unaffected controls. Western blots with mitochondrial protein were probed for a covalent modification caused by HNE, a reactive product of ROS-triggered lipid peroxidation. Overall damage was assessed by densitometry of whole lanes. Data were normalized to reference samples which were present on each blot. Note that damage in the degeneration prone regions brainstem (BS) and cerebellum (CB) of the KO does not exceed the damage in the respective regions from controls. * denotes significant difference after Holm Bonferroni correction at the α = 5% level.

Dysfunctional respiration can lead to excessive production of ROS [26, 27] and, if not adequately detoxified, ROS can cause cell death [28]. In order to assess ROS damage in the CNS of KO animals, we probed mitochondrial protein for a stable end-product of the reaction of 4-hydroxy-2-nonenal (HNE) with lysyl residues [21, 29] (Fig 4). Generally in brainstem and cerebellum more HNE-modified protein was found than in “rest” brain. However KO samples from either young or old brainstem did not markedly accumulate damage in excess of what was found in their healthy genomic controls, neither did old KO samples from cerebellum. It is therefore unlikely that regional imbalance of ROS production/clearance is responsible for focal neurodegeneration.

#### Neuron Specific Respiration

**Fig 5:**
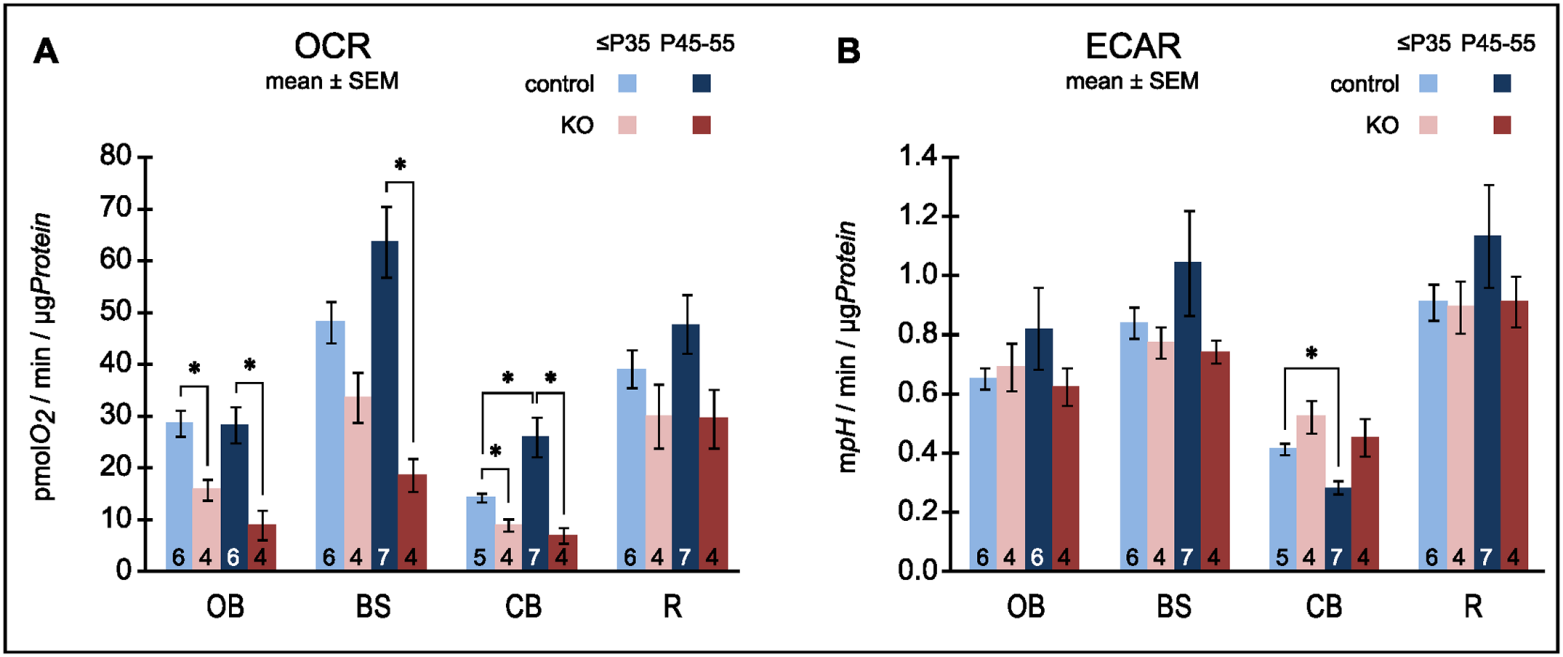
Metabolic capacity of synaptosomes. A: Synaptosomes were fuelled with glucose plus pyruvate and mitochondrial respiration was maximized by uncoupling with the protonophore FCCP. Oxygen consumption rates (OCR) were corrected for non-mitochondrial oxygen consumption. Note, the degeneration-prone regions of the KO: olfactory bulb (OB), brainstem (BS) and cerebellum (CB),—but not the resilient “rest” of the brain (R) -, have significantly lower capacities to respire than those of the controls. B: Glycolytic capacity was assessed as the external acidification rate (ECAR) measured after blocking oxidative phosphorylation and stimulating ATP turnover with 4-aminopyridine. Note, for any given brain region the capacity for glycolysis was fairly stable between genotypes and with age. The only significant (α = 5%) change observed was a decrease with age in the controls (*) from cerebellum. The number of biological repeats is stated inside the bars. * denotes significant (α = 5%) differences after Holm-Bonferroni correction for multiple comparisons.

To determine mitochondrial activity exclusively from neurons, we tested respiration of isolated synaptosomes. The capacity for mitochondrial respiration was determined as the rotenone- plus antimycin A-sensitive FCCP-uncoupled respiration rate with glucose plus pyruvate as external energy substrates. We compared rates between genotypes and age groups (Fig 5A). In the older cohort respiration capacities in the KO were significantly lower in OB, BS, CB than in controls, while the difference did not reach significance for the resilient “rest” brain (R). When normalized to the rates in the control synaptosomes at the late time point (P45-55), the maximal rates of KO synaptosomes from all 3 vulnerable regions were about 1/3 that of controls. In contrast, the resilient KO “rest” brain retained 2/3 of the control activity. In general, we observed a tendency of declining capacity with age in the KO and increasing capacity in controls. It reached significance only in control CB.

#### Neuron Specific Glycolysis

When oxidative phosphorylation is impaired glycolytic ATP production may increase to meet energy demand. Lactic acidosis is a common symptom in LS and upregulation for glycolysis has been reported for the *Ndufs4*(KO) [24]. We assessed the glycolytic capacity of brain region specific synaptosomes by measuring external acidification rates (ECAR) after blocking respiration with rotenone plus antimycin A and further stimulating ATP turnover with 4-aminopyridin, a blocker of the Kv1 (Shaker, KCNA) family of voltage-activated K^+^ channels. Foremost, we did not find compensatory upregulation of glycolytic capacity in the KO in general (Fig 5B). No differences were noted between genotypes for any region or age group (Fig 5B) (with the sole exception of a small decrease in control synaptosomes from cerebellum.)

**Fig 6:**
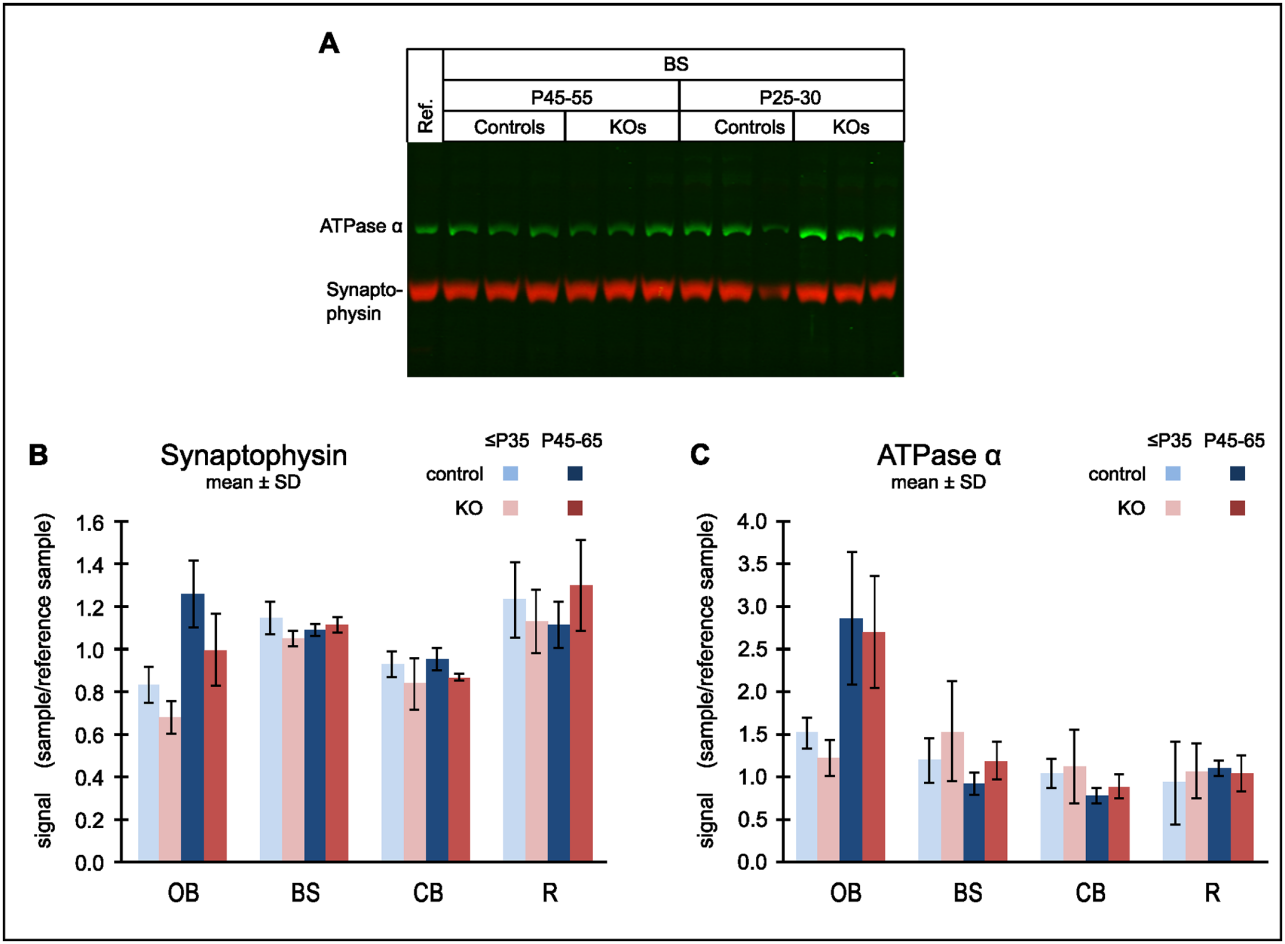
Markers for synaptosomes and mitochondria in synaptosomal preparations. Western blots were simultaneously probed for synaptophysin (red) and the ATPase-α (green). A blot with 3 independent brainstem (BS) synaptosome preparations for each combination of genotype and age group is shown as an example (A). 14 additional blots (not shown) were evaluated to cover samples from this and the other brain regions. The same “rest” brain (R) synaptosome preparation (Reference) was present on each blot for normalization. Densitometry results for synaptophysin (B) and ATPase-α (C): The signal of each band was normalized to the reference signal from the same blot. Data are mean ± SD, N = 3 independent biological samples for each condition with 3 technical repeats per sample.

We considered that regional disease conditions, such as inflammation, might affect the quality of synaptosomal preparations. Monitoring the synaptosomal marker synaptophysin by Western blot we found its relative abundance to be unaffected by either genotype or age in brainstem, cerebellum and “rest” brain. In the olfactory bulb an increase with age was noted (Fig 6A and 6B). Thus, the observed decline in respiratory capacity of KO synaptosomes from the vulnerable regions (OB, BS, CB) cannot be attributed to a parallel decline in preparation purity. Another possible cause for the observed respiratory deficiency in the KO synaptosomes could be depletion of mitochondria from the nerve terminals rather than progressive catalytic failure of complex I. Using ATPase-α, a subunit of ATP synthetase, as a marker for mitochondria, we did not find a meaningful correlation between marker content and respiratory capacity (Fig 6C).

#### Synaptosomal ROS

**Fig 7:**
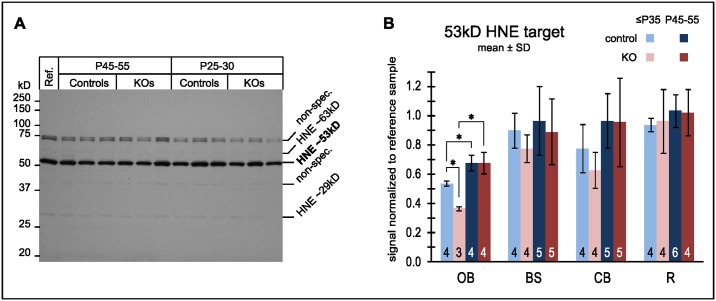
HNE-damage to synaptosomal protein from vulnerable regions of the KO does not exceed damage in controls. ROS damage to synaptosomes was assessed by SDS PAGE Western blots probed for HNE reaction products. A blot (A) with 12 independent brainstem samples is shown as an example. (See supplemental S3 Fig. for the full set.) The positions of the 3 most immunoreactive bands are labeled (HNE) showing an unidentified band at 53kD band as the predominant reaction product. Non-specific bands (non-spec.) were independent of the primary antibody. Densitometry results (B) for all brain regions: For each sample the 53kD signal was normalized to the reference sample (“Ref”), an identical aliquot of which was run on each blot. The number of biological replicates is stated at the bottom of each bar. * denotes significant (α = 5%) differences after Holm-Bonferroni correction for multiple comparisons.

Having found differing regional patterns for synaptosomal and non-synaptosomal capacities for respiration and state3 respectively, region specific ROS damage at the synapse may explain selective loss of respiratory capacity in synaptosomes from the vulnerable brain regions. HNE damage to protein was measured as a proxy for ROS damage (Fig 7A). Olfactory bulb was the only region that significantly accumulated HNE damage (Fig 7B) with age both in KO and controls. However, the final amount did not exceed the fairly constant amount of damage observed in all other regions that was also independent of genotype. In particular no evidence was found to link region specific decrease in metabolic capacity in the KO to abnormal HNE damage.

## Discussion

Our goal was to determine the underlying cause for region-specific tissue damage in a mouse model of Leigh Syndrome.

The data show that global CNS complex I-dependent oxphos capacity was decreased in *Ndufs4*(KO) mice compared to wild-type for pyruvate/malate and glutamate/malate but not for α-ketoglutarate/malate (Fig 1). For state 3 respiration with α-ketoglutarate/malate we consider that both metabolites are imported (independently) into the mitochondria where α-ketoglutarate is metabolized via the TCA cycle to malate which can be exchanged for external α-ketoglutarate. Imported malate is oxidized to oxaloacetate and then decarboxylated to exportable PEP by mitochondrial PEP carboxykinase [30, 31]. The degradation of both substrates is linked by GTP produced by succinyl-CoA synthetase and consumed by PEP carboxykinase [32]. This model is based on pilot experiments (data not shown) showing that (1) supplementing α-ketoglutarate with malate increased state 3 respiration and (2) blocking complex II with malonate could inhibit state3 respiration by not more than 30% (while rotenone completely blocked respiration). These findings were incompatible with a more simple model assuming strict antiport of α-ketoglutarate and malate where external malate would be expected to act as an inhibitor by competing with α-ketoglutarate for uptake. Under our model utilization of α-ketoglutarate/malate produces one FADH2 at complex II for every two NADH. This is in contrast to the other complex I dependent substrates where complex II is either dispensable (pyruvate/malate) or bypassed altogether (glutamate/malate). Thus, α-ketoglutarate maximizes the contribution of complex II to state 3 respiration and vice versa poses the lowest demand on residual complex I activity. This may explain why state 3 rates did not differ between the genotypes.

KO cells would energetically benefit twofold from increased utilization of α-ketoglutarate. First, it would support oxidative phosphorylation by shifting a considerable proportion of the electron influx into the respiratory chain from complex I to complex II. Second, it would utilize substrate-level phosphorylation. Sgarbi et al. [33] rescued ATP synthetase deficient cells by supplementation of the culture medium with a combination of α-ketoglutarate and aspartate. Rescue was attributed to reversal of the malate/aspartate shuttle allowing sustained mitochondrial substrate level phosphorylation without net production of NADH. The same treatment should be even more beneficial for ndufs4 KO cells because here complex II would drive oxidative phosphorylation (which was impossible in the ATP synthase deficient cells). It is therefore tempting to speculate that survival of KO brain regions may be governed by their ability to mobilize beneficial substrates such as succinate or α-ketoglutarate either alone or in combination with aspartate (or malate). Precursors of α-ketoglutarate such as glutamate or glutamine may also be beneficial if enough α-ketoacids are also present as aminogroup acceptors. Moreover, LS may be treated by supplementation of these metabolites.

So far we have no information about the regional levels of α-ketoglutarate. Anaplerotic synthesis of α-ketoglutarate requires the enzyme pyruvate carboxylase (pcx) which is missing in neurons but present in astrocytes [34, 35]. If α-ketoglutarate is neuroprotective one would expect a positive correlation between pcx expression and neuronal survival in the KO. In situ hybridization results, accessible via the Allen Mouse Brain Atlas [36], however do not support this idea.–e.g. the extremely vulnerable olfactory bulb robustly expresses pcx while in thalamus and midbrain, both constituents of the resilient “rest” brain, expression is sparse[36].

In non-synaptic mitochondria from the KO the decrease in complex I activity was similar in both affected and unaffected regions of the brain (Fig 1B, 1C and 1D). In addition, their oxphos capacity did not decrease over time in any of these regions. Hence, the respiratory properties of non-synaptic mitochondria cannot explain region specific neurodegeneration.

In the environment of isolated mitochondria, complex I without NDUFS4 was stable enough to retain considerable residual enzymatic activity but not stable enough to remain assembled when challenged by the presence of the mild detergent digitonin used for BNGs (Fig 2**)**. Thus, the subunit is necessary for the stability of complex I proper, rather than as a docking protein to integrate otherwise stable complex I into supercomplexes. HNE damage was increased in brainstem and cerebellum which are vulnerable in the KO (Fig 4). Since however the same regions in the controls experienced a similar degree of damage without any ill effect on cell survival, regionally elevated ROS stress did not seem to be sufficient to explain regional neurodegeneration.

When focusing on the respiratory capacity of purely neuronal mitochondria by employing synaptosomes, a different picture emerged. The respiratory capacity of the KO synaptosomes was lower in cerebellum, brainstem and olfactory bulb than in the corresponding regions of the wild-type CNS. By contrast, for the “rest” of the brain the difference between the genotypes did not reach statistical significance. Thus, consistent with our hypothesis, we measured diminished respiratory capacity for all the degeneration-prone regions but not for the resilient “rest” brain. It is important to note that in the vulnerable regions of young *Ndufs4*(KO) respiratory capacity was already moderately diminished before the onset of spongiform lesions. Low respiratory capacity is therefore not a trivial consequence of neurodegeneration but cannot be ruled out as its cause. Furthermore, the control animals tended to increase respiration capacity in their nerve terminals with age which may indicate increasing metabolic demand. The KOs on the other hand decreased capacity leading to an increasingly severe energetic deficiency. As noted in the Results, at the late time point (P45-55) the maximal rates of KO synaptosomes from all 3 vulnerable regions were about 1/3 that of controls, while the KO “rest” brain retained 2/3 of the control activity.

For other neurodegenerative diseases involving mitochondrial dysfunction evidence has been reported that neurodegeneration starts with the loss of synapses and axons before it progresses to degeneration of the soma[37, 38]. These results suggest that insufficient energy supply for the synapses may trigger neurodegeneration and disease. A recent study of the bioenergetics of synaptic boutons identified endocytosis of the synaptic vesicles as particularly vulnerable to energy deficiency [39]. Thus, region-specific differences between energy demand at the synapse and capability to meet the demand may explain why neurons in the olfactory bulb and parts of the brainstem and in the cerebellum fail while neurons in other parts of the brain can survive.

It seems curious that wild-type nerve terminals from cerebellum can function properly with only 1/3-1/2 of the respiratory capacity of those from brainstem. However substantial differences between synaptosomes from various brain regions have been noted before and are therefore likely to reflect adaptations to tissue specific energy demand rather than preparation artifacts. The McMurray group investigated wild-type synaptosomes from cerebellum, cortex, striatum and hippocampus and also found cerebellum to be by far the least active [40]. Our respiration rate for cerebellum was however 3 to 5 times higher than their reported activity. The discrepancy in reported rates may simply reflect our choice of pyruvate in addition to glucose as external substrates.

Besides oxidative phosphorylation by mitochondria, aerobic glycolysis in the cytoplasm is the other major source of ATP regeneration. Under circumstances when oxidative phosphorylation is insufficient the capacity for aerobic glycolysis will become critical for cell survival. Johnson *et al*. have reported elevated levels of glycolytic metabolites in KO brain extracts suggesting the possibility of an upregulation of glycolysis as a compensatory mechanism [24]. However, when we measured flux (ECAR) we did not find evidence of increased capacity for glycolysis in region specific samples. Neither did we find decreased capacity compounding the mitochondrial energy deficiency in the KO for any region.

Insufficient respiratory spare capacity has been proposed as a major reason for the death of wild-type neurons [18]. ATP demand for each neuron may fluctuate with stochastically occurring spikes. Neurons die as soon as individual demand exceeds individual capacity. A neuron with high spare capacity can survive random big spikes and only will succumb to extremely unlikely”freak" spikes. Thus, high spare capacity predicts long survival. *Vice versa*, the lower the spare capacity the higher the chance for an early cell death because even the more frequently occurring less severe spikes exceed the threshold. This model nicely accounts for progressive CNS failure as encountered in normal aging as well as in mitochondrial disease. Regionally insufficient spare respiratory capacity provides a possible explanation of the phenomenon of region specific neurodegeneration. It remains however to be discovered how the primary defect, systemic absence of the NDUFS4 protein, brings about insufficient capacity in distinct regions of the CNS.

Assuming the neurons in the various vulnerable regions of the KO brain die for the same reason and by the same mechanism we are under way to investigate regional transcriptomics and metabolomics hoping to find a signature unique to the affected regions which would help to explain their vulnerability and might even lead to a rationale treatment of LS.

In conclusion, our data indicate that in neurons of the vulnerable brain regions of this mouse model of LS respiratory capacity progressively worsens at the synaptic terminals. In contrast, in the part of the brain not developing lesions, respiration capacity is maintained at near normal levels. The inability of vulnerable regions to meet increasing energetic demand at the synapses over time likely leads to local degeneration and progression of disease. The prospect to optimize the use of residual complex I activity by increasing the availability of α-ketoglutarate offers a tantalizing possibility for alleviation of the complex I insufficiency.

## Supporting Information

S1 Fig Complex I independent capacity for oxidative phosphorylation of non-synaptic brain mitochondria. Intact whole brain mitochondria were supplied with the complex II electron donor succinate and the complex I inhibitor rotenone and the ADP stimulated respiration (state 3) was measured. Note: Electron transport capacity downstream of complex I is not limiting complex I dependent state 3 respiration in the KO. The number of biological replicates is given inside the bars. The apparent increase from the younger KO to the older KO did not reach significance (α = 5%) when the Holm Bonferroni correction for multiple comparisons is applied. The same is true for the difference between old KO and controls.
(TIFF)

S2 Fig HNE-damaged protein in non-synaptic mitochondria. Western blots were probed for HNE-damaged mitochondrial protein from brainstem (A), cerebellum (B) and “rest” brain (R). The sample designation indicates the age group (y for P25-P35, o for P45-P55), the genotype (KO, Ctrl for controls) and a number to distinguish independent samples. The HNE signal strength obviously decreased with the sample age (storage duration) indicated below the blots. Therefore in order to allow comparison within a blot/region samples 7 months and older were normalized to oCtrl1 from the same blot while samples 2 months and younger were normalized to oCtrl2. “Ref.” is a reference sample to normalize data between blots/regions. It is always “rest” brain mitochondria from oCtrl4. Whole lane densitometry from A, B, C is the basis for the histogram shown in Fig 2. D is the blot from panel A reprobed for the mitochondrial marker ATPase (ATP5a) to demonstrate that extended sample storage did not degrade sample protein in general. E: Subsequent nonspecific staining with Coomassie Blue 350R (E) [41] confirmed comparable loading and banding patterns independent of sample age. The rightmost two lanes in C were probed with secondary antibody only, to demonstrate that signals shown in A,B and the main part of C are specific to the primary antibody alone. Black lines in the MW lanes are magic marker on the film to indicate the positions of the prestained molecular weight standards on the blot.
(TIFF)

S3 Fig HNE-damaged proteins in synaptosomes. Western blots were probed with anti HNE-fluorophore antibody (see methods for details). The positions for the three most prominent immuno-reactive bands are labeled with their estimated molecular weights. Pilot blots (not shown) indicated that additional bands labeled “non-spec.” did not depend on incubation with the primary antibody. Each sample lane in A-F represents an independent biological sample. Brain region of origin, mouse age group and genotype for each sample are indicated within the panels. In F and D abbreviations OB, BS, CB, R are used for the brain regions: Reference sample, “Ref.”, is always the same “rest” brain P45-55 control sample, run in duplicate on each blot in order to allow normalization of data for comparison between blots. The compiled densitometry results for the predominant immuno-reactive band at approximately 53kD are shown in Fig 7B.
(TIFF)
