# Supplementary figures and images for: Region-Specific Defects of Respiratory Capacities in the Ndufs4(KO) Mouse Brain
Source: PLoS One. 2016 Jan 29;11(1):e0148219. doi: 10.1371/journal.pone.0148219 (PMC4732614; doi:10.1371/journal.pone.0148219)

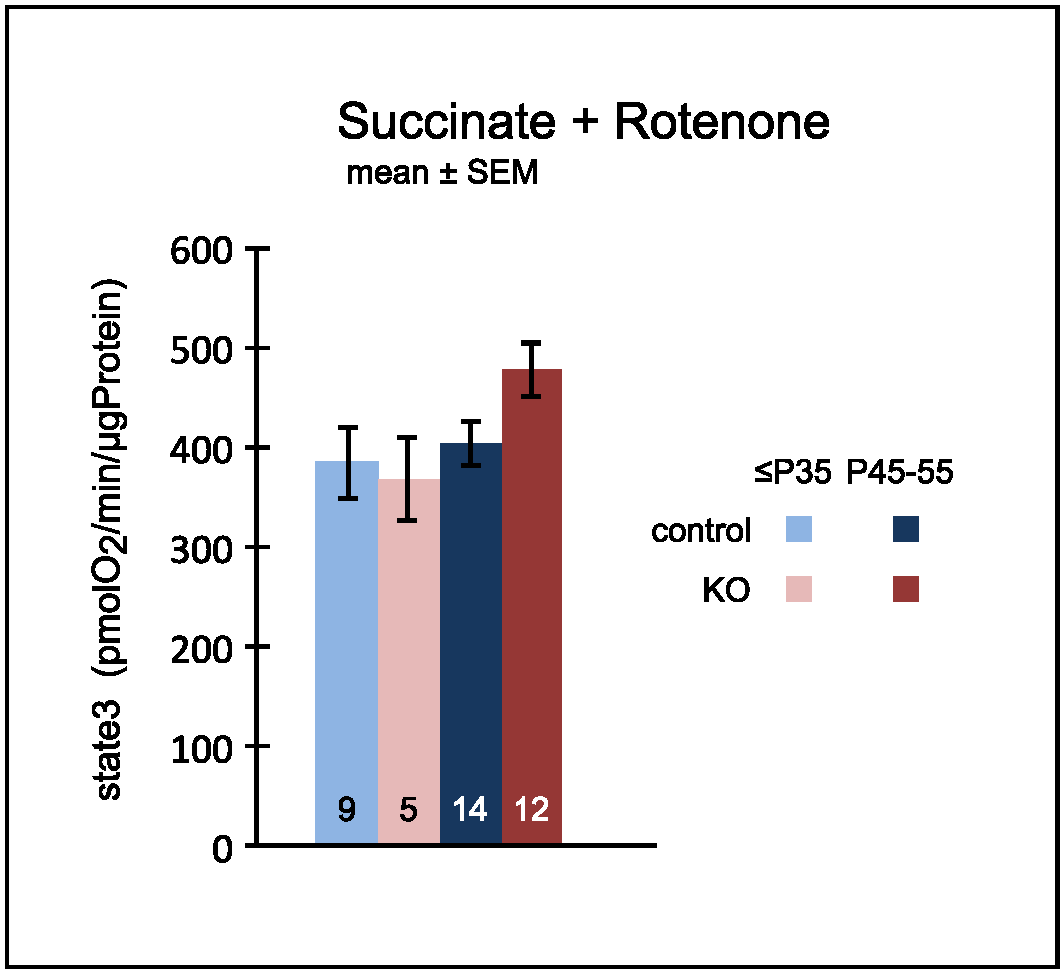

Supplement: S1 Fig — Intact whole brain mitochondria were supplied with the complex II electron donor succinate and the complex I inhibitor rotenone and the ADP stimulated respiration (state 3) was measured. Note: Electron transport capacity downstream of complex I is not limiting complex I dependent state 3 respiration in the KO. The number of biological replicates is given inside the bars. The apparent increase from the younger KO to the older KO did not reach significance (α = 5%) when the Holm Bonferroni correction for multiple comparisons is applied. The same is true for the difference between old KO and controls. (TIFF) [file pone.0148219.s001.tiff]

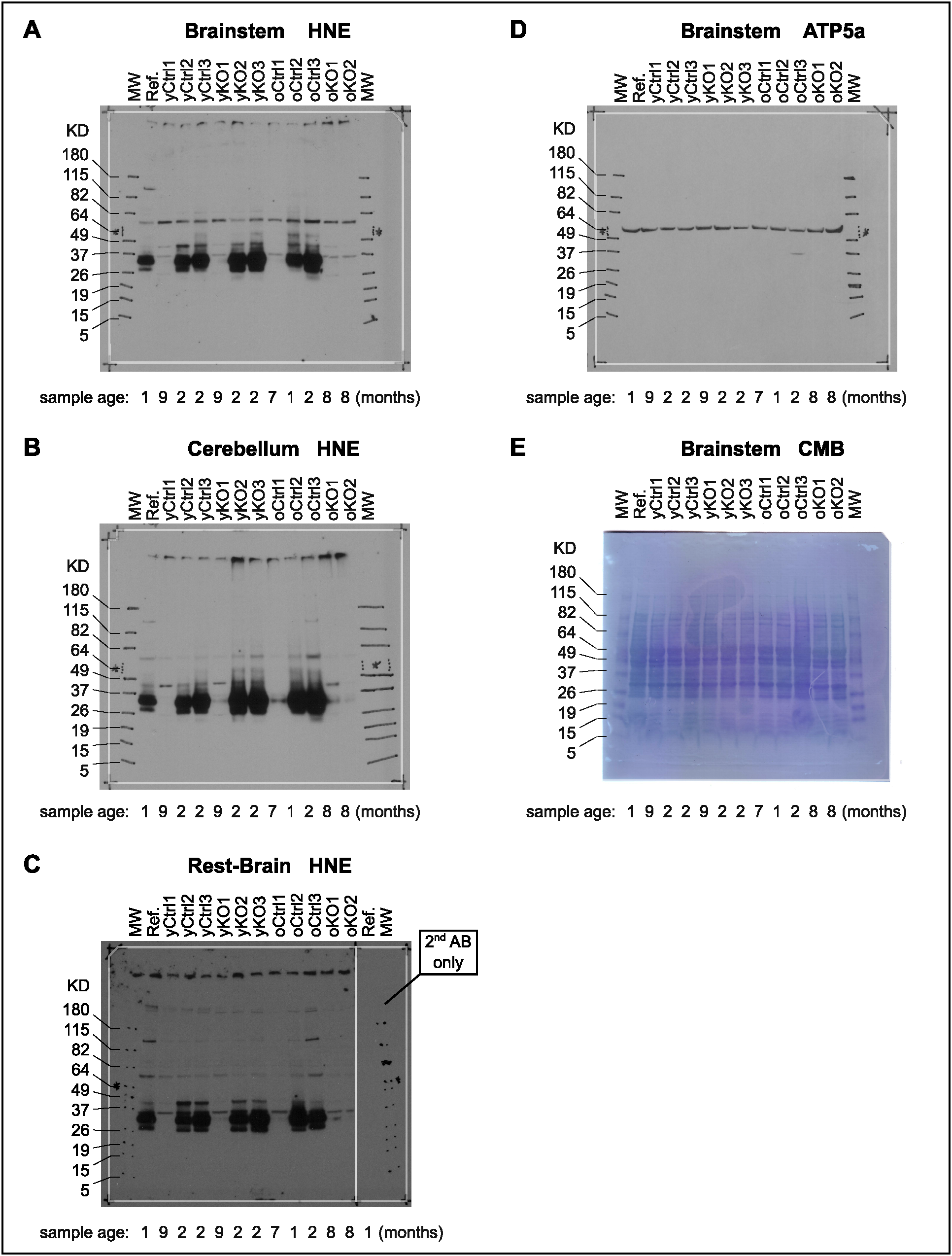

Supplement: S2 Fig — Western blots were probed for HNE-damaged mitochondrial protein from brainstem (A), cerebellum (B) and “rest” brain (R). The sample designation indicates the age group (y for P25-P35, o for P45-P55), the genotype (KO, Ctrl for controls) and a number to distinguish independent samples. The HNE signal strength obviously decreased with the sample age (storage duration) indicated below the blots. Therefore in order to allow comparison within a blot/region samples 7 months and older were normalized to oCtrl1 from the same blot while samples 2 months and younger were normalized to oCtrl2. “Ref.” is a reference sample to normalize data between blots/regions. It is always “rest” brain mitochondria from oCtrl4. Whole lane densitometry from A, B, C is the basis for the histogram shown in Fig 2. D is the blot from panel A reprobed for the mitochondrial marker ATPase (ATP5a) to demonstrate that extended sample storage did not degrade sample protein in general. E: Subsequent nonspecific staining with Coomassie Blue 350R (E) [41] confirmed comparable loading and banding patterns independent of sample age. The rightmost two lanes in C were probed with secondary antibody only, to demonstrate that signals shown in A,B and the main part of C are specific to the primary antibody alone. Black lines in the MW lanes are magic marker on the film to indicate the positions of the prestained molecular weight standards on the blot. (TIFF) [file pone.0148219.s002.tiff]

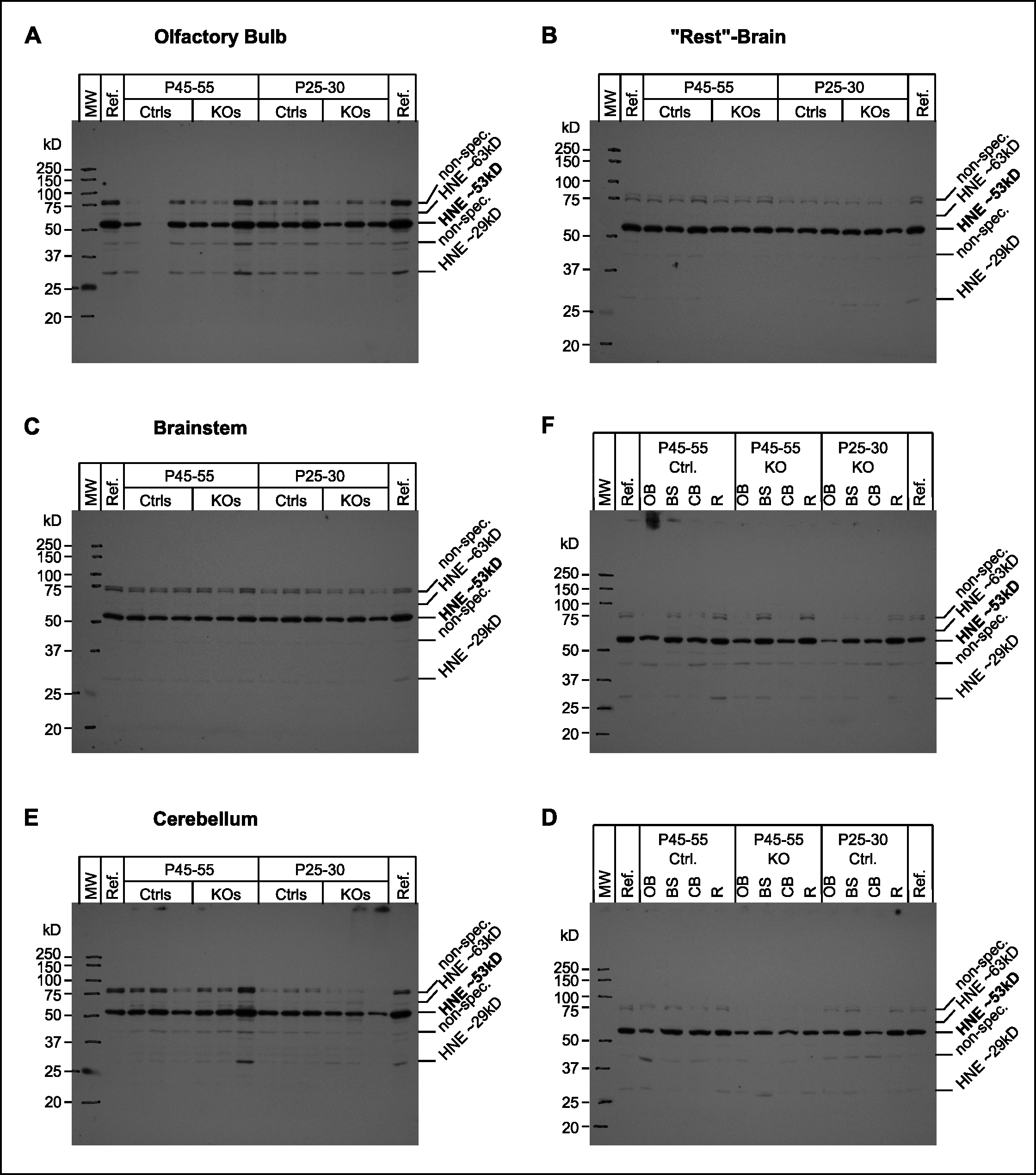

Supplement: S3 Fig — Western blots were probed with anti HNE-fluorophore antibody (see methods for details). The positions for the three most prominent immuno-reactive bands are labeled with their estimated molecular weights. Pilot blots (not shown) indicated that additional bands labeled “non-spec.” did not depend on incubation with the primary antibody. Each sample lane in A-F represents an independent biological sample. Brain region of origin, mouse age group and genotype for each sample are indicated within the panels. In F and D abbreviations OB, BS, CB, R are used for the brain regions: Reference sample, “Ref.”, is always the same “rest” brain P45-55 control sample, run in duplicate on each blot in order to allow normalization of data for comparison between blots. The compiled densitometry results for the predominant immuno-reactive band at approximately 53kD are shown in Fig 7B. (TIFF) [file pone.0148219.s003.tiff]
